# Green Process for Industrial Waste Transformation into Super-Oxidizing Materials Named Alkali Metal Ferrates (VI)

**DOI:** 10.3390/ma12121977

**Published:** 2019-06-19

**Authors:** Ndue Kanari, Etleva Ostrosi, Cécile Diliberto, Inna Filippova, Seit Shallari, Eric Allain, Frederic Diot, Fabrice Patisson, Jacques Yvon

**Affiliations:** 1 GeoRessources Laboratory, UMR 7359 CNRS, CREGU, Université de Lorraine, 2, rue du doyen Roubault, BP 10162, 54505 Vandoeuvre-lès-Nancy, France; inna.filippova@univ-lorraine.fr (I.F.); ericgallain@gmail.com (E.A.); frederic.diot@univ-lorraine.fr (F.D.); jacques.yvon@univ-lorraine.fr (J.Y.); 2 Ville de Montréal, Direction de l’environnement, Division de la Planification et du Suivi Environnemental, 801, rue Brennan, Montréal, QC H3C 0G4, Canada; etlevao@yahoo.com; 3 Institut Jean Lamour, UMR 7198 CNRS, Université de Lorraine, Equipe ‘Matériaux pour le Génie Civil’, IUTNB, BP 90137, 54600 Villers-lès-Nancy, France; cecile.diliberto@univ-lorraine.fr; 4 Agricultural University of Tirana, Faculty of Agriculture and Environment, 1029 Tirana, Albania; seitshallari@gmail.com; 5 Institut Jean Lamour, UMR 7198 CNRS, Labex DAMAS, Université de Lorraine, Campus Artem, 2 allée André Guinier, BP 50840, 54011 Nancy, France; fabrice.patisson@univ-lorraine.fr

**Keywords:** industrial waste, alkali ferrates, super-oxidizing materials, fluidized bed, green process

## Abstract

The investigation presented here features the design of a cleaner and greener chemical process for the conversion of industrial wastes into super-oxidizing materials. The waste of interest is the iron sulfate heptahydrate (FeSO_4_·7H_2_O) mainly generated through the sulfate route used for titanium dioxide industrial production. The products of this transformation process are alkali ferrates (A_2_FeO_4_, A = Na, K) containing iron in its hexavalent state and considered as powerful oxidants characterized by properties useful for cleaning waters, wastewaters, and industrial effluents. The proposed process includes two steps: (i) The first step consisting of the pre-mixing of two solids (AOH with FeSO_4_·xH_2_O) in a rotary reactor allowing the coating of iron sulfate in the alkali hydroxides through solid–solid reactions; and (ii) the second step involves the synthesis of alkali ferrates in a fluidized bed by oxidation of the single solid obtained in the first step in diluted chlorine. The chemical synthesis of alkali ferrates can be carried out within a timeframe of a few minutes. The usage of a fluidized bed enhanced the energy and mass transfer allowing a quasi-complete control of the ferrate synthesis process. The alkali ferrate synthesis process described here possesses many characteristics aligned with the principles of the “green chemistry”.

## 1. Introduction

Iron compounds are abundant in most nonferrous metal deposits where these metals often represent a small fraction. Although part of the ferrous components are separated during mining and primary mineral processing, a large part of iron goes through the extractive processes of these metals. Further, the iron is sometimes present in the natural mineral bodies (e.g., FeTiO_3_, CuFeS_2_, (Ni,Fe)_9_S_8_) of target metals that can be separated during the primary metal extraction. Therefore, considerable amounts of iron bearing co-products and wastes are inevitably generated from both hydro- and pyro-metallurgical operations on such raw materials.

One typical industrial example of generation of iron-containing waste is the extraction of titanium oxide (TiO_2_) from its bearing materials such as ilmenite, rutile, anatase, and slags. As described previously [1], “chloride” and “sulfate” routes are processes currently used for TiO_2_ production. The sulfate process, using ilmenite (FeTiO_3_) as a raw material, can lead to the generation of amounts of iron sulfate (FeSO_4_·7H_2_O—melanterite) as high as 6 tons of FeSO_4_·7H_2_O per ton of produced TiO_2_ [2], which is a real environmental drawback for the TiO_2_ industry. By 1993, all countries producing TiO_2_ through the sulfate process had to comply with the European directive [3] in order to avoid dumping industrial waste (such as melanterite) into the seawater. In the perspective of a circular economy and sustainable development, the ideal scenario should be to consider these wastes (e.g., iron sulfate) as an input for new chemicals and material synthesis, which are intrinsically non-hazardous, thus excluding the notion of undesirable by-product. Few recent investigations [4,5,6,7] address the usage of melanterite in various application and its treatment, such as for synthesizing slow-release fertilizers [5], cation-substituted LiFePO_4_ [6], and for Fe_3_O_4_ production through reductive decomposition using pyrite [7].

As a continuation of our previous research investigations [2,8,9,10], the present work aims at transforming industrial iron sulfate into alkali metal ferrates (A_2_FeO_4_, A = Na, K) considered as useful materials in different fields. One may note that the denomination “ferrate” is generally attributed to compounds containing iron at an oxidation state higher than Fe(III) and the ferrates(VI) seem to be the best known and most studied [2]. In an aqueous solution, the ferrate ion (FeO_4_^2−^) is reduced, generating both Fe(OH)_3_ and nascent oxygen (Equation (1)). As summarized early [2], ferrates are used for water treatment due to their powerful oxidizing capacity (E(FeO_4_^2−^/Fe^3+^ = 2.2 V)) (oxidation of organic and mineral materials, bactericide agent) and because of the flocculating property of the evolved Fe(OH)_3_. The ferrates can replace chlorine in the pre-oxidation stage of water and can partially be used as a substitute of the iron and aluminum salts used as coagulating and flocculating agents. Furthermore, the decomposition of alkali ferrates generates basic medium favorable for the precipitation of heavy metals. These properties existing together in one single compound make ferrates an ongoing material particularly interesting for water treatment and effluent cleaning. Moreover, the oxidation products (iron oxy/hydroxides) (Equation (1)) are inoffensive to the environment.

2FeO_4_^2−^ + 5H_2_O → 2Fe(OH)_3_ + 4OH^−^ + 3O^•^ (1)

The research works performed by Fremy [11,12,13] are frequently cited in the literature as the first ones to scientifically reveal the existence of iron in a hexavalent state and to effectively achieve ferrates synthesis. Since then, the preparation methods, developed mostly on a laboratory scale, have made little progress, and can be classified into three groups:

- The high temperature route consisting of heating and/or melting various iron oxides bearing materials under high concentration of alkali substances and oxygen flow [14,15,16]. These synthesis methods, performed at temperatures as high as 800 °C, seem to be mostly ineffective since the Fe(VI) is not stable at temperatures higher than 200 °C. Most probably, the Fe(VI) resulted from the dismutation of synthetized Fe(IV) and/or Fe(V) during the manipulation with the synthesis product.
- The wet/humid oxidation of Fe(III) salts bearing solutions, under strong alkaline conditions, using hypochlorite or chlorine as oxidant. This method is the most used since the 1950s [17,18,19,20]. One of the drawbacks is that the wet method used pure chemicals and required many operations for Fe(VI) preparation and separation, making it very costly. Moreover, the water reacts with ferrate (Equation (1)) leading to its reduction into Fe(III).
- The electrochemical method [21,22,23], allowing the oxidation of anode (iron or its alloys) by operating mainly in concentrated NaOH and/or KOH solutions. However, the decomposition of Fe(VI) by water, low current efficiency, and anode passivation are some of the concerns for this route.

Synthesis of alkali ferrates (AF) by gas–solid reactions performed in a rotary reactor using chlorine as an oxidant showed that synthesis was achieved without external heat supply [2,8,9,10]. However, it was observed that the synthesis reactions were highly exothermic leading to a temperature rise in the reaction zone above 150 °C, when only 10 g of solids were used for the potassium and/or sodium ferrate synthesis. Experimental results indicated that the heat generation provoked the sample agglomeration and dramatically decreased the Fe(VI) efficiency of the synthesis process. The temperature increase phenomena became a real “obstacle” when higher amounts (100 g) of solids were used for the AF synthesis, although the reactor was cooled. Further, the kinetic of Na-ferrate synthesis is expected to be low compared with that of potassium ferrate [2]. In other words, it was concluded that the synthesis of alkali ferrates in a rotary reactor would not be considered as an appropriate route for an eventual AF large-scale production.

In this context, the goal of this research work is threefold: (i) Use the industrial iron sulfate as raw material for the AF synthesis; (ii) develop an appropriate process for AF preparation at room temperature; (iii) optimize the process by data analysis of various parameters affecting the efficiency of Fe(II,III) conversion into Fe(VI). The AF synthesis (mostly sodium ferrate preparation) is achieved in fluidized bed (FB) which can be considered as an appropriate reactor for handling processes that require high energy and mass transfers. It should be noted that the alkali metal ferrates (VI) manufacturing process, as developed in this work, is unique in its field.

## 2. Materials and Methods

Industrial ferrous sulfate (mainly monohydrate) is used for the synthesis of alkali ferrates. An examination by scanning electron microscope (Hitachi Ltd., Tokyo, Japan) coupled with energy dispersive spectrometry (Kevex Corp., Foster, CA, USA) (SEM-EDS), X-ray diffraction (XRD), chemical analyses, and Mössbauer spectroscopy (MS) suggested that the sample is free of heavy metals and that the quasi-totality of iron is in a bivalent state as FeSO_4_·H_2_O. Another sample of ferrous sulfate heptahydrate is also used after dehydration in an oven at about 150 °C leading to the formation of FeSO_4_·H_2_O and FeSO_4_·OH as the main iron-bearing phases. Commercial sodium hydroxide is used as pellets of 2 mm and pearls of about 1 mm. The oxidizing agent (chlorine) and diluting gas (nitrogen) were of high purity, whilst the air was supplied by a compressor.

**Figure 1:**
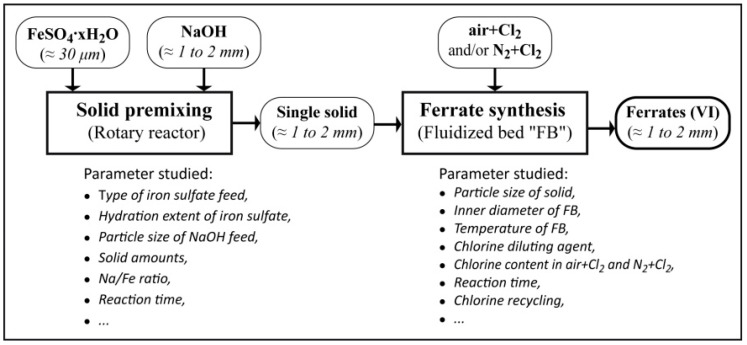
Schematic representation of the experimental procedure applied for the ferrates(VI) synthesis through a two-step process.

**Figure 2:**
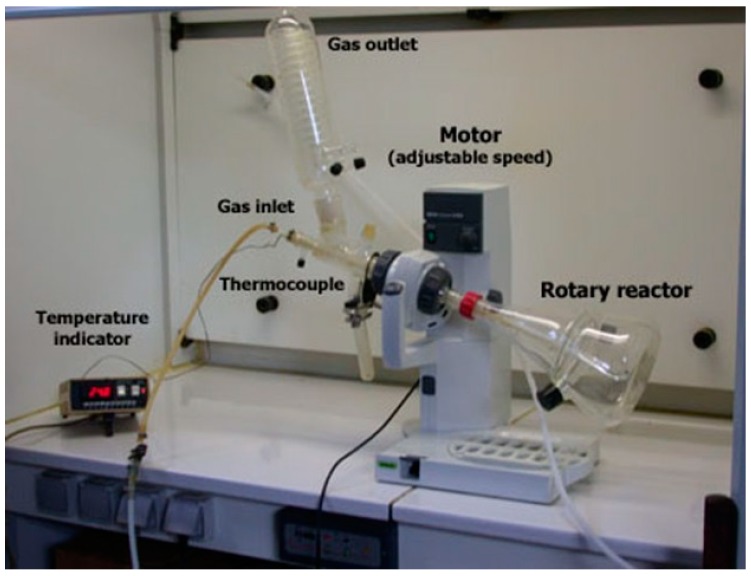
Apparatus assembly with a baffled reactor used for mixing iron sulfate with sodium hydroxide.

The flowchart summarizing the features of the experimental protocol established for the synthesis of alkali ferrates is schematized in Figure 1. Accordingly, the synthesis process consisted of two main steps. The first step consists of a premixing of mostly FeSO_4_·H_2_O and/or FeSO_4_·OH with NaOH resulting in a single solid. This step is realized by using the experimental setup represented in Figure 2. The parameters studied for the solid premixing step are related to physical characteristics of iron sulfate and NaOH feed, their amounts, as well as the premixing time.

The second step of the synthesis process (Figure 1) consists of the reaction of the granulated and single solid, prepared in step 1, with chlorine diluted in air and/or N_2_ leading to the sodium ferrate preparation. A fluidized bed reactor equipped with a temperature-regulated water system is used for the experimental tests of alkali ferrate synthesis. A second fluidized bed is added in series for recycling the unreacted oxidant (chlorine) during AF synthesis taking place in the first fluidized bed. Several experimental parameters related to the ferrate synthesis step (particle size of solid, temperature, chlorine content, reaction time) were investigated. Other details of the experimental procedure will be introduced when describing the experimental results for both steps.

Solid synthesis products are subjected to visible microscopy, SEM-EDS, and XRD to examine the structure and the composition of the solid reaction products. The procedure of these characterization techniques is given in a recently published material [24].

Mössbauer spectroscopy was used to seek information about the oxidation state of iron as well as to evaluate the Fe(VI) content in the synthesis product. Details about this analysis method were given earlier by Jeannot et al. [25]. However, this method of examination is time consuming, i.e., the acquisition of a Mössbauer spectrum may take up to 24 h. In this context, a chemical analysis method is performed to quickly determine the Fe(VI) synthesis efficiency of the ferrate synthesis trials. This method is based on the chemical reaction of Fe(VI) with an excess of ferrous sulfate solution; then, the excess of Fe(II) is titrated with potassium bichromate.

## 3. Results and Discussion

### 3.1. Concept of the Vibrating Fluidized Bed

**Figure 3:**
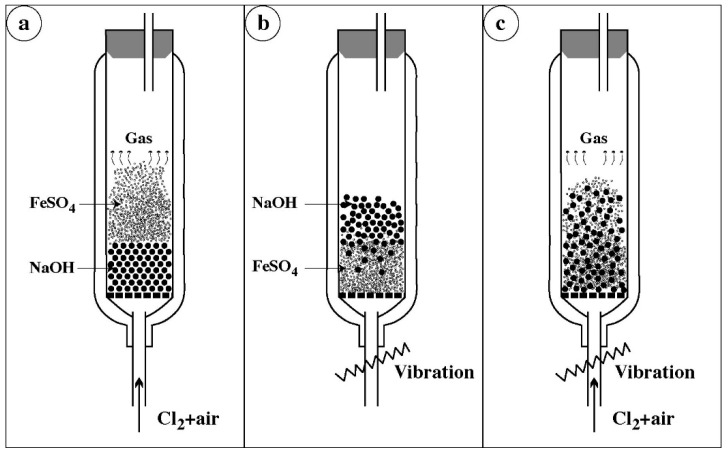
Schematic particles distribution of solids in a vibrated fluidized bed: (**a**) optimal gas fluidization velocity for iron sulfate; (**b**) only vibration of fluidized bed; (**c**) simultaneous blowing and vibration of fluidized bed.

The fluidized bed technique is very attractive for different processes related to gas–solid reactions and for its easy extrapolation. However, a fluidized bed is not suitable for solids with important differences in particles sizes (or density), as it is the case of NaOH and iron sulfate. As a reminder, the mean particle sizes of NaOH, found in market, were about 1, 2, and 5 mm and that of FeSO_4_·H_2_O is less than 100 μm. A conventional fluidization of NaOH and FeSO_4_·H_2_O (for a given fluidization velocity) allows a heterogeneous distribution of the solids in the reactor. It was suggested that the vibrating fluidized bed would be a solution for the homogeneous fluidization of NaOH and iron sulfate. The distribution of particles in the bed through the respective conditions of the fluidization alone, vibration alone, and fluidization coupled with vibration are schematized in Figure 3. The choice of the optimal fluidization velocity of the reactive gases for the iron sulfate creates the distribution situation described in Figure 3a. Thus, iron sulfate is fluidized when the NaOH pearls creates a packed bed at the bottom of the reactor. If the bed is only vibrated (Figure 3b), the pearls of NaOH move towards the top, while the iron sulfate accumulates at the bottom of the reactor. Meanwhile, the use of blow and vibration leads to an almost homogenous ‘fluidization’ of both solids (FeSO_4_ and NaOH) as described in Figure 3c. However, it seems that the vibrating fluidized bed is difficult to control on an industrial scale production, especially for delicate processes such as ferrate synthesis.

### 3.2. Idea of Premixing of Solids Prior to Fluidization

The overall reaction of the sodium ferrate synthesis can be described by Equation (2), although the exact formula of this compound seems to be still undefined. It was already confirmed experimentally that the synthesis of Na-ferrate via Equation (2) is exothermic. The possible “two by two” reactions of the three substances (FeSO_4_·H_2_O, Cl_2_, and NaOH) could be represented by Equations (3)–(5). Iron sulfate monohydrate does not react with chlorine at room temperature. Recent experience in the field of gaseous chlorine reactivity with iron compounds showed that the oxidation of Fe(II) of wüstite (FeO) into Fe(III) takes place at temperatures higher than 200 °C [26]. Chlorine in the presence of oxygen can oxidize Cr(III) into Cr(VI), generating chromium oxychloride [27,28,29].

As could be expected, the reaction of chlorine with NaOH produces NaCl as a final reaction product involving heat with ∆H = −128 kJ/mol NaOH [30]. The most interesting reaction to be considered is that of iron sulfate with sodium hydroxide (Equation (5)) resulting in the formation of Na_2_SO_4_. The sodium sulfate is also an unavoidable product of the Na-ferrate synthesis (Equation (2)). Consequently, it was suggested to react iron sulfate with NaOH, allowing them to form a single solid (mixture of Fe(OH)_2_ and Na_2_SO_4_), which would be suitable for the subsequent fluidization in the FB.

The following paragraphs will describe the experimental results of the solid premixing concept.

FeSO_4_·H_2_O + 2Cl_2_ + 8NaOH → Na_2_FeO_4_ + Na_2_SO_4_ + 4NaCl + 5H_2_O (2)

FeSO_4_·H_2_O + Cl_2_ → No evident reaction at room temperature (3)

2NaOH + Cl_2_ → 2NaCl + H_2_O + 0.5O_2_ (4)

FeSO_4_·H_2_O + 2NaOH → Fe(OH)_2_ + Na_2_SO_4_ + H_2_O (5)

### 3.3. Premixing of NaOH Pellets (2 mm) with Iron Sulfate

**Figure 4:**
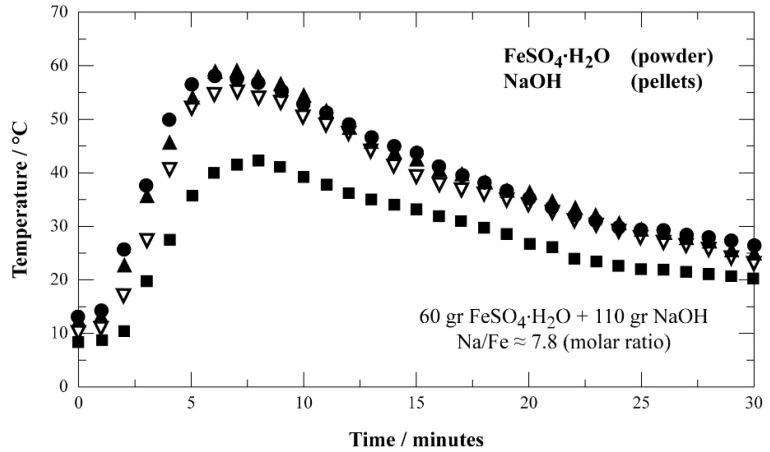
Evolution of the temperature as a function of time for the reaction of FeSO_4_·H_2_O during their mixing in the rotary reactor (four trials run in same conditions).

Sodium hydroxide conditioned as 2-mm pellets was used for the experimental tests. Iron sulfate monohydrate was chosen as iron salt for the same tests. The reaction of these solids was carried out in a rotary reactor of 2.5 L without presence of any gas and using an apparatus of premixing step as illustrated in Figure 2. About 170 g of solids with a molar ratio Na/Fe close to 8 (to satisfy Equation (2)) were loaded in the reactor and rotated at a speed of 20 rpm. The premixing time was fixed at 30 min. The evolution of temperature during the reaction was recorded and the data are displayed as plots of temperature versus time in Figure 4.

**Figure 5:**
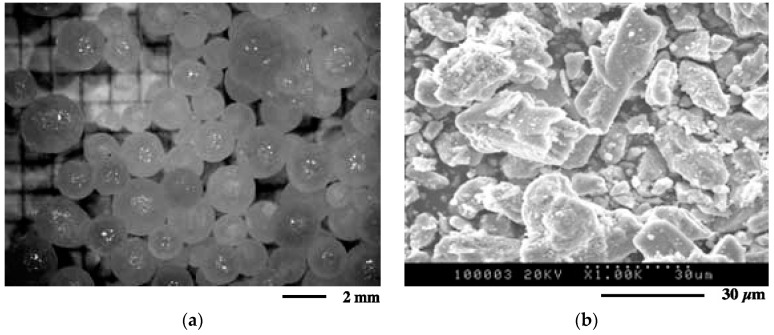
View of sodium hydroxide and ferrous sulfate samples: (**a**) Visible microscopy (VM) images of NaOH pellets; (**b**) SEM images of FeSO_4_·H_2_O.

As shown by this figure, the reaction is exothermic leading to a temperature of about 60 °C in the reaction zone. The NaOH pellets become gray-black, but they more or less keep their initial shapes. Images of initial substances NaOH pellets (visible microscopy (VM)) and FeSO_4_·H_2_O (SEM) are shown in Figure 5a,b, respectively. Figure 5a clearly shows that NaOH consists of spherical particles of a diameter lower than or equal to 2 mm. As a contrast, FeSO_4_·H_2_O is composed of grains of different shapes with an equivalent diameter lower than 30 μm (Figure 5b).

**Figure 6:**
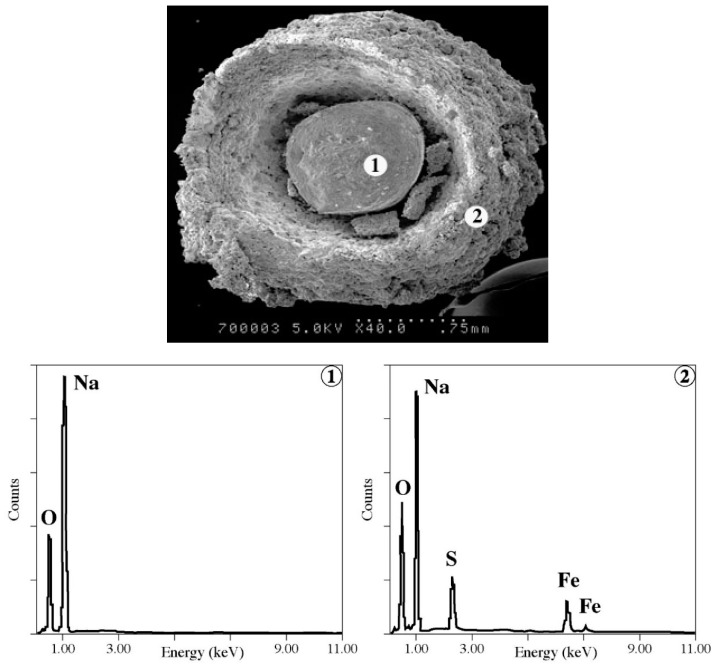
Scanning electron microscope coupled with energy dispersive spectrometry (SEM-EDS) examination results of a NaOH pellet reacted with FeSO_4_·H_2_O: (1) core of the NaOH pellet; (2) outer part of the NaOH pellet.

Some relevant information about the reaction of NaOH with FeSO_4_·H_2_O was revealed by SEM-EDS investigation. A representative NaOH grain after reaction with iron sulfate (image SEM) and its elemental analyses (EDS) are grouped in Figure 6.

The examination of these results suggests the following deductions:

- The external part of the NaOH pellets (spectrum 2) is essentially composed of Na, O, S, and Fe, indicating the “reaction” of NaOH with iron sulfate,
- The NaOH pellets become porous (photo of Figure 6) facilitating the diffusion of reactive gases (chlorine) during the Na-ferrate synthesis,
- The core of the pellets is composed of Na and O showing the presence of unreacted NaOH (note that the amount of NaOH was 4 times more than the stoichiometry of the reaction: 2NaOH + FeSO_4_ → Fe(OH)_2_ + Na_2_SO_4_).

XRD analysis showed the presence of NaOH and NaOH·H_2_O as predominant crystallized phases in the mixture. The iron-bearing phase was not revealed by XRD. However, the chemical analysis of the obtained mixture indicated that this mixture contained about 93 g Fe/kg and that 54% of iron was in a three-valence state. The presence of Fe(III) was also confirmed by Mössbauer spectroscopy measurements.

These pre-mixing materials were subjected to the Na-ferrate synthesis in a fluidized bed using diluted chlorine as an oxidation reagent.

### 3.4. Preliminary Fluidization Tests of the Premixing Materials in the Fluidized Bed

As it is well known, the fluidization of a solid by gas depends on the physical characteristics of the solid such as the density, particle size, shape, and those of the gas (gas viscosity and density).

The Reynolds (*Re_MF_*) and Archimedes (*Ar*) numbers as well as the minimum velocity of fluidization (*U_MF_*) are calculated by using the relationships available in the literature. The correlation of Wen and Yu [31] is used in this work, which is considered as the most known relationship related to a narrow particle size distribution. Equations (6)–(8) describe the formulas for calculating *Re_MF_*, *Ar,* and *U_MF_*.

Reynolds number at minimum fluidization (*Re_MF_*):

(6) $$Re_{MF}={\left[33.7^{2}+0.0408\frac{d_{p}^{3}\rho_{g}\left(\rho_{s}-\rho_{g}\right)g}{\mu_{g}^{2}}\right]}^{1/2}-33.7$$

where *μ_g_* is gas viscosity (Pa·s(kg·s^−1^·m^−1^)); *ρ_g_* is gas density (kg·m^−3^); *ρ_s_* is particle density (kg·m^−3^); *d_p_* is particle size (m); and *g* is gravitational constant (m·s^−2^).

Archimedes number (*Ar*):

(7) $$Ar=\frac{d_{p}^{3}\rho_{g}\left(\rho_{s}-\rho_{g}\right)g}{\mu_{g}^{2}}.$$

Minimum velocity of fluidization (*U_MF_*):

(8) $$U_{MF}=Re_{MF}\frac{\mu_{g}}{d_{p}\rho_{g}}$$

The numeric substitution of the (*μ_g_*, *ρ_g_*, *ρ_s_*, *d_p_*, *g*) values showed that the mean minimum velocity of the fluidization of the premixing materials is about 1 m·s^−1^. However, these values are approximate ones. For example, the solid density is considered to be 2130 kg·m^−3^ (density of NaOH), but it will be less if we consider that the NaOH particles become porous during the reaction with iron sulfate (see photo of Figure 6).

Based on these calculations, an FB of an internal diameter of about 1.75 cm was designed and constructed for the synthesis of Na-ferrate by using the NaOH conditioned as 2 mm pellets.

For the Na-ferrate synthesis, about 10 g of the premixed solids were loaded in the FB, creating a column of 9 to 10 cm. A total gas flow rate of 1100 L/h was necessary to ensure the fluidization of solids. The chlorine content of the used air + Cl_2_ and N_2_ + Cl_2_ gas mixtures was kept at 5.5%, whilst the synthesis time was fixed at 15 min. The temperature of the thermostated water varied from 25 to 55 °C.

**Figure 7:**
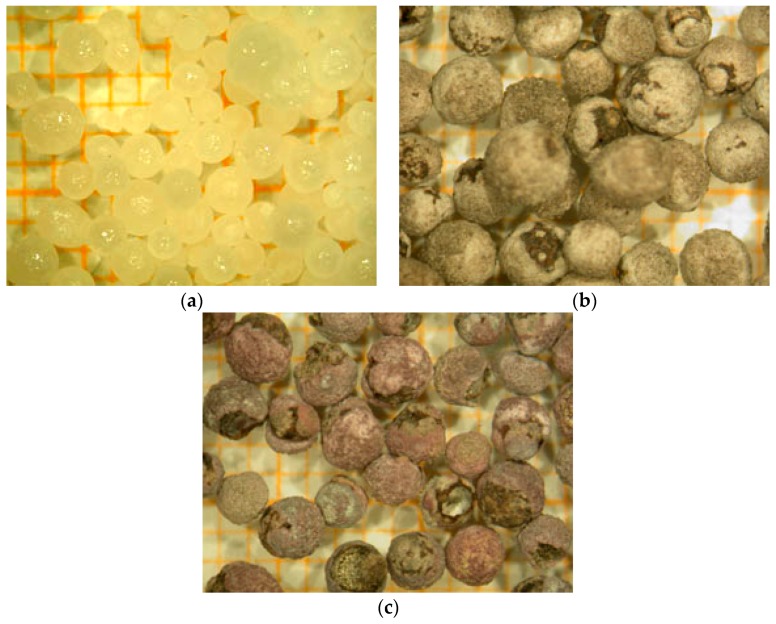
Visible microscopy images of NaOH-pellets at different steps of the process (the small graduation of the paper in the background is 1 mm large). (**a**) Initial state of NaOH; (**b**) NaOH + FeSO_4_·H_2_O (premixing); (**c**) NaOH + FeSO_4_·H_2_O + Cl_2_ (fluidized bed).

Visual observations indicated a good fluidization of the solids in FB without dust formation. This reinforces the idea that iron sulfate was well cemented during premixing (NaOH + FeSO_4_·H_2_O) in the rotary reactor. The solids after treatment in FB were examined by VM. The images of VM are compared with those of NaOH and premixing solids as shown in Figure 7. The results confirmed that the solids before reaction with chlorine (Figure 7b) and after (Figure 7c) had similar obvious shapes. The purple color of the solid surfaces suggested the formation of Na-ferrate. Furthermore, the dissolution in water of the solids coming from FB gave evidence of iron presence in its hexavalent state. The experimental conditions, as well as the chemical analysis of products issued from the synthesis process, are summarized in Table 1.

The synthesis products contained about 10 to 21 g/kg of iron in a hexavalent state. The best results were obtained when nitrogen was used as diluting gas. This is probably due to the presence of moisture in the air, which can decompose the synthesized ferrate. A higher Fe(VI) yield was achieved when the water bath was regulated at 55 °C. These preliminary tests showed the possibility of the Na-ferrate synthesis in a fluidized bed using the mixture (NaOH + FeSO_4_·H_2_O) as raw materials. However, as the NaOH-pellets of 2 mm are no longer found in the market, it is suggested to test the synthesis of Na-ferrate using NaOH-pearls of about 1 mm (1000 μm).

### 3.5. Premixing of NaOH Pearls (1000 μm) with Iron Sulfate

The tests carried out with pearls of sodium hydroxide and monohydrated ferrous sulfate in a reactor of 2.5 L (see Figure 2) showed that these reagents did not react like the NaOH pellets of 2 mm. Therefore, it was suggested to add FeSO_4_·7H_2_O in order to initiate the reaction. One may note that the particle size of FeSO_4_·7H_2_O is about 0.7 mm.

**Figure 8:**
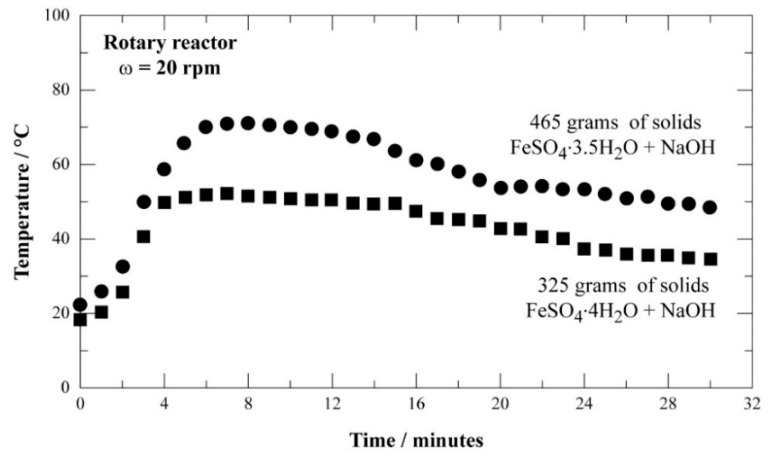
Evolution of the temperature as a function of time during the mixing of NaOH with iron sulfate (≈0.7 mm) containing 3.5 and 4 mol of water.

Figure 8 is a typical example of measured temperatures versus time in case of using ferrous sulfate with 3.5 and 4 mol of water (mixture of FeSO_4_·7H_2_O and FeSO_4_·H_2_O).

**Figure 9:**
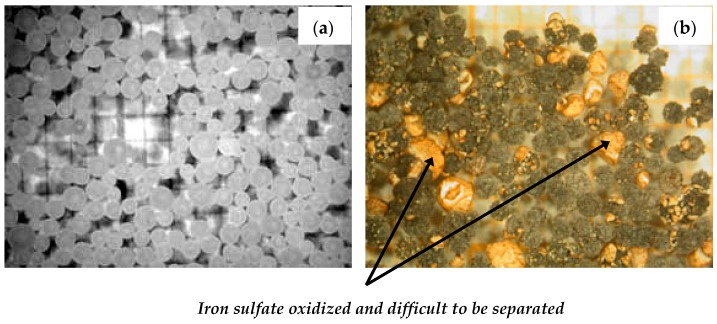
Visible microscopy images of sodium hydroxide (pearls-1 mm): (**a**) Initial state; (**b**) after reaction with FeSO_4_·4H_2_O (≈0.7 mm).

The molar ratio Na/Fe was fixed at 8 (to satisfy Equation (2) for the subsequent ferrate synthesis). This figure indicates that it was possible to mix about 325 and 465 g by using a hydration degree of 4 and 3.5 mol for the iron sulfate, respectively. The maximum temperature in the reactor did not exceed 80 °C. However, a close examination of the obtained mixture revealed that iron sulfate heptahydrate had not fully reacted with NaOH. This situation is presented in Figure 9 (visible microscope). Figure 9a showed the initial state of NaOH pearls, while Figure 9b represents the VM view of the mixture. As shown by Figure 9b, a large amount of FeSO_4_·7H_2_O is oxidized, agglomerated, and was difficult to separate by sieving.

**Figure 10:**
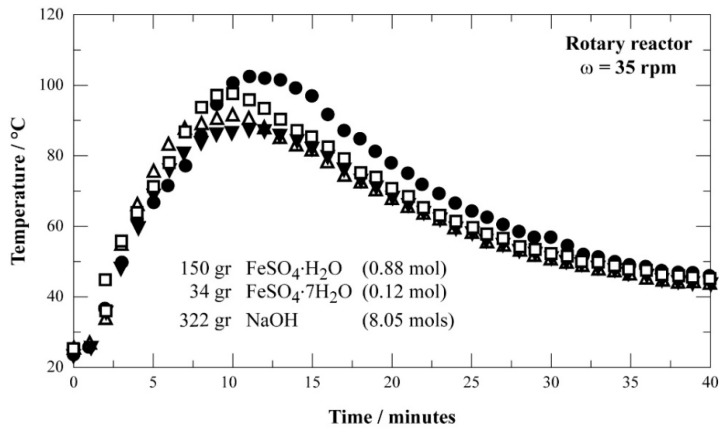
Evolution of the temperature as a function of time during mixing of NaOH with iron sulfate (powder) containing about 1.74 mol of water (four trials run in same conditions).

To overcome the phenomenon observed above, it was planned to use iron sulfate heptahydrate in powder form. The use of 0.12 mol FeSO_4_·7H_2_O in powder form, 0.88 mol FeSO_4_·H_2_O, and 8 mol NaOH provided the best results for obtaining the mixture (NaOH + FeSO_4_·7H_2_O + FeSO_4_·H_2_O). The results of several solid premixing tests are plotted in Figure 10, as the evolution of reactor temperature against reaction time in the case where about of 500 g of solids are used. As it could be expected, the reaction was exothermic, and the maximum temperature oscillated between 80 and 100 °C. The output mixture was sieved at +850 μm and was used for the synthesis of Na-ferrate in a fluidized bed.

**Figure 11:**
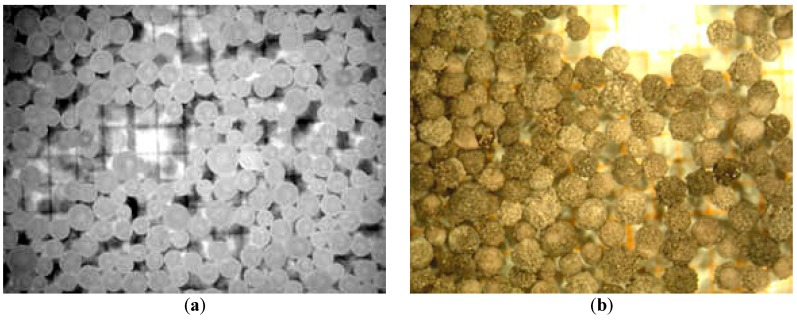
VM views of sodium hydroxide at: (**a**) Initial state, ≤1 mm; (**b**) After reacting with powder iron sulfate.

Figure 11a,b compares the VM images of the initial state of NaOH pearls and that of the obtained mixture according to trials mentioned above. The obtained mixture had a morphology similar to the initial NaOH particles.

### 3.6. Synthesis of Na-Ferrate in Various Fluidized Beds

A fluidized bed with a cross section of about 7.07 cm^2^ (Ø = 3 cm) was used for the synthesis of Na-ferrate based on the NaOH-pearls mixed previously with iron sulfate. It was interesting to follow the evolution of the temperature inside the fluidized bed as the reaction progressed; this was performed by placing a thermocouple in the fluidized bed and recording the temperature.

**Figure 12:**
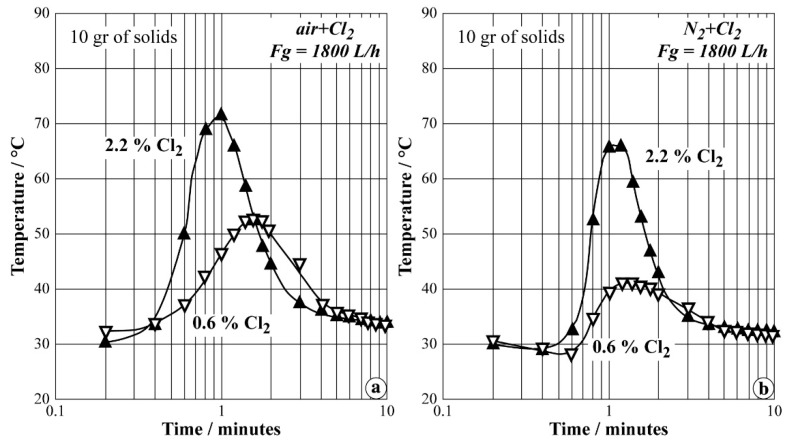
Plots of temperature evolution versus time during the synthesis of Na-ferrate in FB (Ø 3 cm) at 35 °C for: (**a**) air + Cl_2_; (**b**) N_2_ + Cl_2_.

About 10 g of prepared mixture were loaded in the FB. Tests were performed at regulated water temperatures varying from 20 °C to 65 °C. The total flow rate of (air + Cl_2_) and (N_2_ + Cl_2_) gaseous mixture was 1800 L/h corresponding to the operational fluidization velocity for the particles in the FB of Ø 3 cm. The data obtained for a water temperature set at 35 °C are plotted in Figure 12a,b when air + Cl_2_ and N_2_ + Cl_2_ are used, respectively. The chlorine contents of both gaseous mixtures were fixed at 0.6% and 2.2% Cl_2_.

These figures clearly show that the temperature inside the FB increased sharply during the first minute of treatment. Use of 2.2% Cl_2_ led to a higher maximum temperature level in both cases. The maximum temperatures observed were slightly lower when nitrogen was used instead of air. These observations are also valid over a water temperature range between 20 °C and 65 °C. The obtained products from these tests were examined by dissolving them in water. It was observed that most of the synthesized product did not show evidence of ferrate presence in the product. Data analyses suggested that the partial pressure of chlorine in the system was too low, making the oxidation of iron at high valence impossible. In order to have the flexibility of supplying high chlorine partial pressure, it was suggested to use a fluidized bed with a smaller diameter.

Tests in the FB with Ø 2 cm were carried out in a similar procedure as described for the previous case scenario. The total gas flow rate was 800 L/h corresponding to the operational fluidization velocity as in the case of FB with Ø 3 cm. The regulation temperature of water was varied from 18 °C to 55 °C, while the chlorine content was fixed at 2.5% and 5.0%. The evolution of the temperature as a function of the reaction time had a shape similar to that observed with FB Ø 3 cm.

The visual tests in water for the obtained product indicated that all the synthesis products at 2.5% chlorine were characterized by the absence of Na-ferrate. As a contrast, the synthesis products at 5.0% chlorine and the water regulated temperature higher than 18 °C showed some evidences of the presence of Na-ferrate. These results confirmed that the Na-ferrate synthesis is strongly affected by chlorine partial pressure.

Several synthesis products were subjected to a chemical analysis to determine the content in total iron and Fe(VI). A summary of the experimental conditions of the Na-ferrate synthesis in FB of Ø 2 cm and chemical analyses results is given in Table 2. These results indicated that the synthesis product (after 5 min of synthesis) contained between 72 and 74 g/kg of iron with about one half of iron as Fe(VI). Furthermore, the use of air instead of nitrogen decreased slightly the Fe(VI) efficiency.

Although the obtained results for the Na-ferrate synthesis efficiency were improved in the smallest fluidized bed (Ø = 2 cm), it was observed that the fluidization regime was better in the FB of Ø = 3 cm. For this reason, the fluidized bed of intermediate diameter (Ø = 2.5 cm) was also checked. The experimental procedures of the corresponding tests are similar to those developed for the FB of 3 and 2 cm.

**Figure 13:**
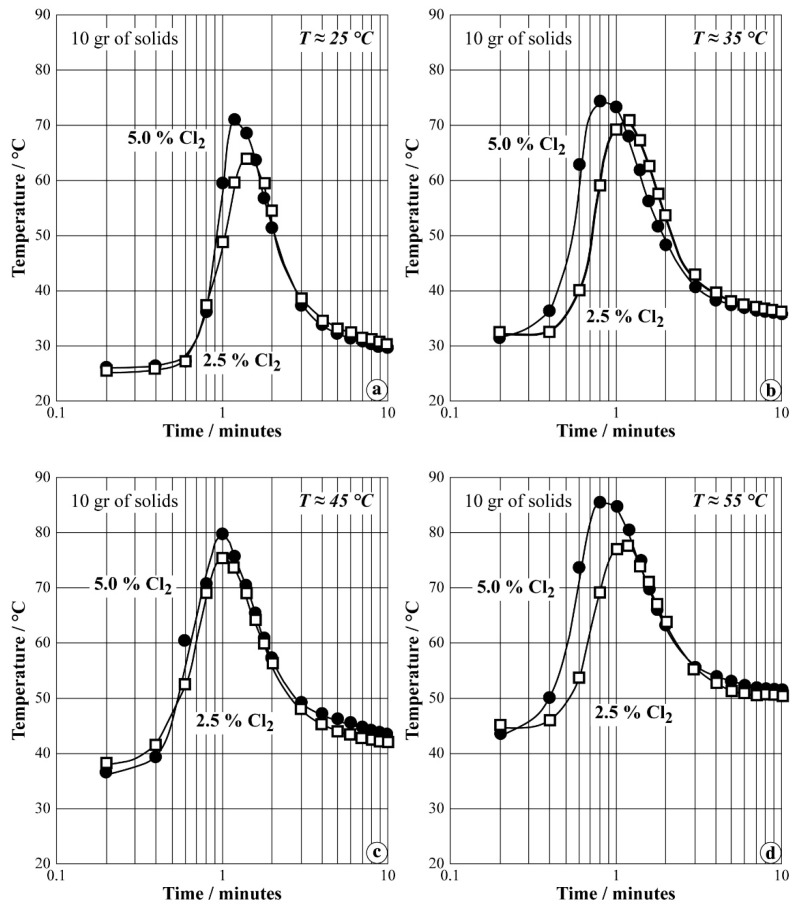
Plots of temperature evolution versus time during synthesis of Na-ferrate in FB (2.5 cm) using air + Cl_2_ for regulated water temperature at: (**a**) 25 °C; (**b**) 35 °C; (**c**) 45 °C; (**d**) 55 °C.

Several experimental results are presented in Figure 13a–d plotted as the evolution of temperature inside the fluidized bed versus the reaction time in a half-logarithm scale. As in the previous case, the synthesis process seems to be completed in 5 min.

Table 3 groups the experimental conditions and chemical analysis for three chosen tests of the whole series of experiments performed with FB of Ø = 2.5 cm. The iron content of the synthesis process is about 75 g/kg, while the Fe(VI) yield varied between 43% and 54%. It seems that the Fe(VI) synthesis efficiency decreased when the regulated temperature exceeded 40 °C. The fluidization regime in FB of Ø = 2.5 cm is better (more homogenous) than in the case of Ø = 2 cm.

### 3.7. Recycling of Non-Reacted Chlorine

In optimum conditions of the fluidized bed tests, the content of chlorine in the gaseous mixtures (air + Cl_2_ and/or N_2_ + Cl_2_) was kept at 5%. As could be expected, only a certain fraction of chlorine is used for the ferrate synthesis. A part of unreacted chlorine was released through off-gases. Attempts were made to recycle this non-reacted chlorine. This was realized by connecting two fluidized beds in series. The off-gases of the first fluidized bed are used to supply the second FB.

About 10 g of the premixing material (single solid) were loaded in each FB. Gas mixture of N_2_ + Cl_2_ (5% Cl_2_), which was introduced in the first FB, also passed through the second FB. A good fluidization of the first FB was observed, while the fluidization of second FB was more difficult, and the material showed a tendency to agglomerate. This is due to the water released during the ferrate synthesis in the first FB and causing a material agglomeration in the second FB. Therefore, it was suggested that the non-reacted chlorine can be recycled after a drying process to eliminate any trace of humidity. This can be easily achieved on an industrial scale, guaranteeing that no gaseous effluent is generated during the ferrate synthesis through the proposed process.

### 3.8. Environmental Considerations of the Process

Although this research is devoted to the preparation of alkali metal ferrates(VI) at room temperature, only some selected literature reports [32,33,34,35,36,37,38,39,40,41,42,43] prove the attractive properties of Fe(VI) for different end-use applications. Adjectives such as “environmentally friendly oxidant”, “green oxidant”, “strong oxidant”, “powerful oxidant”, and “super-iron battery” are often reserved for this class of materials.

The synthesis of ferrates, as described by this process, should be considered as a “green chemistry” process. The following paragraphs list some supporting evidence of this by comparing the principles of green chemistry [44] with our suggested process.

- Atom economy—synthetic methods should be designed to maximize the incorporation of all materials used in the process into the final product (almost all materials used are found in the final synthesis product);
- Safer solvents and auxiliaries—the use of auxiliary substances should be made unnecessary wherever possible and innocuous when used (no solvent or auxiliary substances—separation agents—are used for the ferrate synthesis by the proposed process);
- Design for energy efficiency—if possible, synthetic methods should be conducted at ambient temperature and pressure (it is exactly the case of the ferrate synthesis by the proposed process);
- Use of renewable feedstocks—a raw material or feedstock should be renewable rather than depleting whenever technically and economically practicable (raw materials used for the ferrate synthesis, i.e., Cl_2_ and NaOH supply, is considered as almost as non-depletable);
- Design for degradation—chemical products should be designed so that at the end of their function, they break down into innocuous degradation products and do not persist in the environment (ferrates are transformed during their usage into hydroxides and/or oxides of Fe(III), considered as environmentally friendly compounds).

## 4. Conclusions

In this research, the possibility of transforming an industrial waste into supper-oxidizing materials (alkali metal ferrates) containing iron in its hexavalent state is shown. The following conclusions may be drawn from this investigation:

The proposed process for the synthesis of alkali ferrates included two main steps: (i) Premixing of NaOH with iron sulfate (solid–solid reactions) leading to a single solid, and (ii) fluidization of the obtained mixture in diluted chlorine (gas–solid reactions).

The premixing of the solids was achieved in a rotary reactor and the overall reaction was exothermic. The number of solids, the NaOH particle size, the Na/Fe ratio, and the moisture of the input materials (FeSO_4_·H_2_O and/or FeSO_4_·7H_2_O) affected this step. Temperatures lower than 100 °C were required to obtain good results for the premixing.

The output materials of the premixing, a single solid, contains about 80 to 90 g of Fe/kg of iron depending on the experimental conditions. Both Fe(II) and Fe(III) are present in the obtained mixture.

Various fluidized beds (with different cross sections) were designed and tested for the fluidization of the already prepared mixture. The fluidization was realized by air + Cl_2_ and/or N_2_ + Cl_2_ assuring operational fluidization velocities.

The synthesis tests are carried out by varying parameters such as temperature of the thermostated water, partial pressure of chlorine, type of NaOH (2-mm pellets and 1-mm pearls), reaction time, etc.

The results suggest that fluidization is easy to achieve and that almost no dust is generated during the Na-ferrate synthesis in the fluidized bed, indicating that the iron sulfate is well embedded in the NaOH grains during the premixing step. The synthesis process is exothermic, and it is completed within a few minutes. Heat and water are rapidly evacuated from the reaction zone, leading to a dried Na-ferrate. This is a substantial advantage of performing the ferrate synthesis in a fluidized bed.

It seems that temperatures regulated close to 30 °C and temperatures in the fluidized bed lower than or equal to 70 °C (due to the exothermic reactions) provide the best results for the Na-ferrate synthesis. The Fe(VI) synthesis efficiency varied between 30% and 55% depending on other experimental parameters.

Ferrates obtained by this process could be used directly for different applications without any additional preliminary treatment (such as crushing), keeping in mind that the mean particle size of the ferrate produced by this invented process is close to 1 mm.

The proposed synthesis process meets numerous green chemistry and sustainable development principles. The synthesis product, alkali metal ferrates(VI), belongs to an advanced materials category with multipurpose functions for water and wastewater treatment as well as for cleaning various industrial effluents.

## 5. Patents

Kanari, N. Method of producing ferrates(VI). French patent, publication date: 14 March 2008, no. 2 905 609. Extension at international level: 13 March 2008, no. WO 2008/029046.

## Figures and Tables

**Table 1:** Experimental conditions and Fe(VI) efficiency of synthesis using NaOH pellets of 2 mm.

Gas Mixture, Flow Rate		Cl_2_ (%)	T (°C)	t (min)	Fe(VI) (g/kg)	Fe(VI) (%)
Air + Cl_2_, L/h	1100	≈5.5	25	15	14.8	20.1
	1100	≈5.5	35	15	15.8	21.2
N_2_ + Cl_2_, L/h	1100	≈5.5	25	15	9.8	12.7
	1100	≈5.5	35	15	14.4	17.6
	1100	≈5.5	45	15	18.4	22.8
	1100	≈5.5	55	15	21.1	28.8

**Table 2:** Experimental conditions and Fe(VI) efficiency of synthesis in FB of Ø = 2 cm.

Gas Mixture, Flow Rate		Cl_2_ (%)	T (°C)	t (min)	Fe(VI) (g/kg)	Fe(VI) (%)
Air + Cl_2_, L/h	800	≈5.0	40	5	36.2	49.3
	800	≈5.0	50	5	36.8	50.3
N_2_ + Cl_2_, L/h	800	≈5.0	40	5	39.7	55.5
	800	≈5.0	50	5	37.5	51.0

**Table 3:** Experimental conditions and Fe(VI) efficiency of synthesis in FB of Ø = 2.5 cm.

N_2_ + Cl_2_ (L/h)	Cl_2_ (%)	T (°C)	t (min)	Fe_Total_ (g/kg)	Fe(VI) (g/kg)	Fe(VI) (%)
1200	≈5.0	30	5	74.6	40.1	53.8
1200	≈5.0	40	5	74.7	39.6	53.0
1200	≈5.0	50	5	74.4	31.9	42.9
